# Transitioning young adults from paediatric to adult care and the HIV care continuum in Atlanta, Georgia, USA: a retrospective cohort study

**DOI:** 10.7448/IAS.20.1.21848

**Published:** 2017-09-01

**Authors:** Sophia A. Hussen, Rana Chakraborty, Andrea Knezevic, Andres Camacho‐Gonzalez, Eugene Huang, Rob Stephenson, Carlos del Rio

**Affiliations:** ^1^ Hubert Department of Global Health, Emory University Rollins School of Public Health, Atlanta, USA; ^2^ Department of Medicine, Division of Infectious Diseases, Emory University School of Medicine, Atlanta, USA; ^3^ Department of Paediatrics, Division of Infectious Diseases, Emory University School of Medicine, Atlanta, USA; ^4^ Department of Biostatistics, Emory University Rollins School of Public Health, Atlanta, USA; ^5^ Department of Health Behaviour and Biological Sciences, University of Michigan School of Nursing, Ann Arbor, USA

**Keywords:** adolescent, transition, HIV, care engagement, continuum, engagement, youth

## Abstract

**Introduction**: The transition from paediatric to adult HIV care is a particularly high‐risk time for disengagement among young adults; however, empirical data are lacking.

**Methods**: We reviewed medical records of 72 youth seen in both the paediatric and the adult clinics of the Grady Infectious Disease Program in Atlanta, Georgia, USA, from 2004 to 2014. We abstracted clinical data on linkage, retention and virologic suppression from the last two years in the paediatric clinic through the first two years in the adult clinic.

**Results**: Of patients with at least one visit scheduled in adult clinic, 97% were eventually seen by an adult provider (median time between last paediatric and first adult clinic visit = 10 months, interquartile range 2–18 months). Half of the patients were enrolled in paediatric care immediately prior to transition, while the other half experienced a gap in paediatric care and re‐enrolled in the clinic as adults. A total of 89% of patients were retained (at least two visits at least three months apart) in the first year and 56% in the second year after transition. Patients who were seen in adult clinic within three months of their last paediatric visit were more likely to be virologically suppressed after transition than those who took longer (Relative risk (RR): 1.76; 95% confidence interval (CI): 1.07–2.9; *p* = 0.03). Patients with virologic suppression (HIV‐1 RNA below the level of detection of the assay) at the last paediatric visit were also more likely to be suppressed at the most recent adult visit (RR: 2.3; 95% CI: 1.34–3.9; *p* = 0.002).

**Conclusions**: Retention rates once in adult care, though high initially, declined significantly by the second year after transition. Pre‐transition viral suppression and shorter linkage time between paediatric and adult clinic were associated with better outcomes post‐transition. Optimizing transition will require intensive transition support for patients who are not virologically controlled, as well as support for youth beyond the first year in the adult setting.

## Introduction

Adolescents and young adults represent a growing number of people living with HIV in the United States, reflecting both improvements in survival among perinatally infected youth and the rising incidence of sexually acquired HIV during adolescence [1,2]. Youth living with HIV (YLHIV) ages 13–24 in the US often enter into HIV care in a paediatric clinical setting before undergoing transition to adult‐oriented healthcare. This transition period has been posited as a high‐risk time for disengagement from care; however, empirical data on this topic are relatively scarce [3,4].

Most of the literature on this topic consists of qualitative studies of transition barriers, expert opinions and case descriptions [4]. These qualitative studies have delineated many potential barriers to transition, including disruption of the patient–paediatric provider relationship, need for youth‐friendly clinic environments and loss of ancillary support services once in adult care (e.g. social workers) [5–7]. As a whole, the body of transition research focuses more on vertically (perinatally) than horizontally (behaviourally) infected youth, despite the fact that the majority of YLHIV in the US acquired infection sexually during adolescence [8]. This discrepancy is due in large part to regional and national differences in normative transition processes. In most European countries, YLHIV are predominantly perinatally infected and are typically transitioned around 18 years of age, and age at transition of vertically infected YLHIV is highly variable across sub‐Saharan Africa, ranging from approximately 13 to 22 years [9,10]. In contrast, transition in the US typically occurs in the mid‐20s, and the majority of transitioning patients have horizontally acquired infection, with a preponderance of these YLHIV being young men who have sex with men [10].

There have been very few previously published studies of transition in HIV that include objective clinical measurements of outcomes post‐transition. Ryscavage et al. retrospectively analysed a cohort of 50 HIV‐infected youth (both vertically and horizontally infected), who had been transitioned to adult care in Baltimore and found high rates of linkage to adult care but low rates of retention at 12‐month follow‐up [11]. Poor retention was noted to occur in spite of well‐delineated protocols that aimed to facilitate healthcare transition in that institution. Higher retention rates were found in single‐centre studies conducted in Italy, Sweden and Thailand; these cohorts were almost exclusively made up of vertically infected youth who were transitioned at younger ages (typically age 18 or younger in the Italian and Thai studies; median age was 19 in the Swedish study) relative to the Baltimore cohort (where the median age at linkage to adult care was 24.5) [12–14]. Weijsenfeld et al. utilized national data sets to retrospectively analyse virological and social outcomes in a sample of 59 YLHIV representing multiple centres in the Netherlands, the majority of whom were perinatally infected and transitioned at 18–19 years of age [15]. The authors found high rates of virologic failure during the period of healthcare transition, particularly among youth with low educational attainment and lack of autonomy regarding their medical care.

One reason for the paucity of data on this topic in the US, in spite of widespread interest, is the often insurmountable challenge of obtaining data from both the paediatric and the adult healthcare settings for a given cohort of patients [16]. The Grady Infectious Disease Program (IDP) clinic in Atlanta, Georgia, is a large, multidisciplinary HIV care facility that is home to separate clinic spaces for paediatric/adolescent and adult‐oriented care. Although the clinics are physically separate, they are located in the same building and share the same electronic medical record (EMR) system, therefore presenting an opportunity to compare patient outcomes in the paediatric and adult settings. Our objective was to evaluate engagement in HIV care before, during and after transition from paediatric to adult care in a retrospective cohort of YLHIV.

## Methods

We conducted a retrospective cohort study among HIV‐infected adults who had been enrolled in both the paediatric and the adult clinics at the IDP clinic at any point between 2004 and 2014. The IDP clinic is entirely dedicated to the care of people living with HIV and serves a low socioeconomic status population, nearly all of whom fall below federally defined poverty levels. The majority of patients are either uninsured or funded by Medicaid; uncovered costs are paid by the Ryan White Care Act. Lack of insurance is therefore not a barrier to receiving care at the IDP facility. The paediatric clinic cares for children from ages 0 through 24; the majority of these patients are horizontally infected and enrol in the clinic as adolescents and young adults. There is currently no uniform protocol in this facility for transitioning patients to adult care; the details of this process vary and are dependent on the individual paediatric provider, adult provider and patient in question. For example, in some cases, paediatric providers may identify an adult provider and arrange either a brief introduction or an entire appointment with the adult provider before the transition officially occurs. In other cases, however, a patient is directly referred to the main clinic for their first adult appointment at the time of discharge from the paediatric programme.

We first identified all patients who had been seen in the paediatric clinic and turned 21–25 (the transition age was previously 21 and is now 25) between 2004 and 2014. Scheduled appointments were reviewed in the EMR; those who never had an adult clinic visit scheduled were excluded from the analysis, as they may have moved or otherwise disengaged from care before becoming eligible for transition, and their care engagement is not necessarily reflective of the transition process. We then abstracted data on linkage, retention and virologic suppression as well as demographic and clinical covariates. To facilitate comparisons, we abstracted all data from the last two years in the paediatric clinic (the two years leading up to the last paediatric clinic visit) through the first two years in the adult clinic (the two years beginning with the first adult clinic visit). This study was approved by the Emory Institutional Review Board and Grady Hospital Research Oversight Committee.

Our engagement outcomes of interest were drawn from HIV care continuum conceptual framework [17], with a focus on *linkage, retention and virologic suppression. Linkage* was defined by time elapsed between the last paediatric visit and the first adult clinic visit. No gold standard for linkage time in transition exists; however, most IDP patients are seen every three to four months; so we examined rates of successful linkage within three and six months of the last paediatric visit. Our definition of *retention* was based on the Institute of Medicine (IOM) definition, which requires completion of at least two visits separated by at least three months within a 12‐month period [18]. We assessed whether or not patients met this metric within the first 12 months after initiating adult care and again two years (within the first 24 months) after initiating adult care. *Virologic suppression* was defined as any value below the limit for the site‐specific assay, which changed slightly over the time period assessed here (all cutoffs were 80 copies/ml or less).

### Statistical methods

All analyses were conducted using SAS v 9.4 (Cary, NC, USA). Demographics, clinical characteristics and primary outcomes of the sample were reported and summarized using descriptive statistics. Change in engagement in paediatric versus adult clinic was assessed by comparing the proportions of patients with retention and virologic suppression in paediatric care versus adult care, using McNemar's test. Characteristics were compared between patients who were enrolled in paediatric clinic immediately before transition and those who experienced a gap in care before re‐enrolling as adults, using two‐sample *t*‐tests, Chi‐square tests and Fisher's exact tests, where appropriate. Multivariable logistic regression was used to determine significant factors related to the three main engagement outcomes (linkage to adult clinic, retention and virologic suppression); covariates included demographic and social factors (sex, race and age) and clinical characteristics (mode of HIV acquisition, baseline CD4 count). Effect sizes for these characteristics are tested with Cohen's *d* statistic for mean comparisons and Cohen's *w* statistic for contingency tables [19].

## Results

### Baseline descriptions

We identified 72 patients who had appointments scheduled in both the paediatric and the adult clinics between 2004 and 2014 (Table 1). The majority were males (62.5%), with a sizable proportion (30.6%) of females and a smaller proportion identifying as transgendered (6.9%). The preponderance of the sample was black/African‐American (93.0%). The most common mode of HIV acquisition was male–male sexual contact (62.5%), followed by heterosexual contact (22.2%) and perinatal infection (15.3%).

**Table 1:** Demographic, baseline medical characteristics and transition of care (*N* = 72)

Characteristics	*N*	Frequency (%) or median (interquartile range)
Gender	72	
Male		45 (62.5)
Transgender female		4 (5.5)
Female		22 (30.6)
Transgender male		1 (1.4)
Race	72	
Black/African‐American		67 (93.0)
More than one race		3 (4.2)
White		2 (2.8)
Means of HIV transmission	72	
Male–male sexual contact		45 (62.5)
Heterosexual		16 (22.2)
Perinatal		11 (15.3)
Insurance status	72	
Uninsured		42 (58.3)
Medicaid		19 (26.4)
Private insurance		11 (15.3)
VL suppressed at time of last paediatric visit	68	26 (38.2)
Age at last paediatric visit	72	23.8 (22.0–24.8)
Age at first adult visit	70	24.8 (23.0–25.2)
CD4 count at time of last paediatric visit (cells/mm^3^)	71	341 (172–553)
Calendar year of last paediatric visit	72	
2004–2007		15 (20.8)
2010–2011		13 (18.1)
2012		19 (26.4)
2013		15 (20.8)
2014		10 (13.9)
Transition of care		
Linked from paediatric care to adult provider	72	70 (97.2)
Linked within three months	72	24 (33.3)
Linked within six months	72	29 (40.3)
Time between last paediatric and first adult visit^a^ (months)	70	10 (2–18)
Transition type^a^	70	
Enrolled in paediatric care immediately prior to transition		35 (50)
Experienced a gap in paediatric care and re‐enrolled in clinic as adult		35 (50)
Retained in first year of adult care^a^ (two visits or more in year after transition)	70	62 (88.6)
VL suppressed after first year of adult care^a^	49	26 (53.1)
Retained in second year of adult care^a^ (two visits or more in second year after transition)	68^b^	38 (55.9)
VL suppressed after second year of adult care^a^	38	19 (50.0)

Values are reported as frequency (%) for categorical variables and median (interquartile range) for continuous variables. ^a^Does not include two patients who never successfully transitioned to adult care. ^b^Two patients did not have sufficient follow‐up time to determine retention at the two‐year time point. VL: viral load.

Median age at the time of final paediatric visit was 24 years (interquartile range (IQR): 22–25). Median age at time of final paediatric visit does not differ statistically by gender or mode of acquisition (Wilcoxon rank‐sum test *p* = 0.72, 0.87, respectively). At the time of their final paediatric visits, 38.2% of patients with available data were virologically suppressed, and the mean CD4 T‐lymphocyte count was 341 (IQR: 172–553). Sixty‐five (92.9%) patients met the IOM definition for retention during their last year being followed in the paediatric clinic.

### Linkage to adult care

A total of 70/72 paediatric patients with appointments scheduled in adult clinic successfully made the linkage and were seen by a provider in adult clinic (Table 1). One‐third were linked within three months, and 40% were linked within six months. The median time between the last paediatric and first adult visit was 10 months (IQR: 2–18). Thirty‐five patients (50%) were enrolled in paediatric care immediately prior to transition, while the other 50% experienced a gap in paediatric care and re‐enrolled in the clinic after reaching the age threshold for adult clinic enrolment.

### Change in engagement in paediatric versus adult clinic

We compared each individual patient's clinical data from the last two years in the paediatric clinic with their data in the first two years in the adult clinic; rates of retention and virologic suppression were compared between the two clinics for each patient (Table 2). There was no significant difference between retention in paediatric clinic and retention one year after entry to adult clinic (95% versus 89%); however, retention was significantly lower two years after initiating adult care (95% versus 56%; *p* < 0.001). Virologic suppression did not differ when comparisons were made of the paediatric and adult settings.

**Table 2:** Change in engagement and outcome in paediatric versus adult clinic (*N* = 70)

	Pre‐transition			*p*^a^
**Post‐entry to adult care ‐ year 1**	No retention	Retention	Total	0.32
No Retention	2	6	8–11.4%	
Retention	3	59	62–88.6%	
Total (%)	5–7.1%	65–92.9%	70	
**Post‐entry to adult care ‐ year 2**	No retention	Retention	Total	<0.001
No retention	4	26	30–44.1%	
Retention	1	37	38–55.9%	
Total	5–7.4%	63–92.7%	68	
**Post‐entry to adult care ‐ year 1**	No VL suppression	VL suppression	Total	0.20
No VL suppression	15	5	20–43.5%	
VL suppression	10	16	26–56.5%	
Total	25–54.4%	21–45.6%	46	
**Post‐entry to adult care ‐ year 2**	No VL suppression	VL suppression	Total	0.44
No VL suppression	11	6	17–48.6%	
VL suppression	9	9	18–51.4%	
Total	20–57.1%	15–42.9%	35	

^a^Results of McNemar's test of paired proportion for paediatric versus adult clinic. VL: viral load

### Planned transition compared with falling out of care

We compared those patients who were enrolled in paediatric care immediately prior to transition to those who experienced a gap in care and later re‐enrolled as adults (Table 3). Those who were enrolled in paediatric care prior to transition were significantly more likely to be virologically suppressed at the last paediatric visit compared to those who experienced a gap in care (52% versus 27%; *p* = 0.04). Additionally, the group that was enrolled in paediatric care prior to transition had a higher rate of retention in the second year of adult care (65% versus 27%), higher rate of viral load suppression at time of last adult visit (54% versus 35%) and higher CD4 count at the last adult visit (mean 406 versus 299); however, these associations did not reach statistical significance (*p* = 0.14, *p* = 0.11 and *p* = 0.15, respectively). The majority of variables show a small to medium effect size when tested with Cohen's statistics (Cohen's *w* values: 0.1–0.2). There was a small effect size for CD4 count at time of last paediatric visit and at time of first adult visit (Cohen's *d*: 0.1, 0.3, respectively). Age at last paediatric visit, age at first adult visit and the linkage time between the two visits showed large effects (Cohen's *d*: 1.2, 0.6 and 2.3).

**Table 3:** Differences between patients who were enrolled in paediatric clinic immediately prior to transition and those who experienced a gap in care and re‐enrolled as adults (*N* = 70)

	Enrolled in paediatric clinic pre‐transition (*n* = 35)	Experienced gap in care pre‐transition (*n* = 35)	*p*^a^	Effect size^b^
No. (%) or mean (SD) [sample size, if observations missing]				
Gender				
Male	24 (68.6%)	20 (57.1%)	0.78	0.2
Transgender male	0	1 (2.9%)		
Female	9 (25.7%)	12 (34.3%)		
Transgender female	2 (5.7%)	2 (5.7%)		
Race				
Black/African‐American	32 (91.4%)	33 (94.3%)	0.61	0.2
More than one race	1 (2.9%)	2 (5.7%)		
White	2 (5.7%)	0		
Means of HIV transmission				
Male–male sexual contact	24 (68.6%)	20 (57.1%)	0.59	0.1
Heterosexual	6 (17.1%)	9 (25.7%)		
Perinatal	5 (14.3%)	6 (17.1%)		
Insurance status				
Uninsured	17 (48.6%)	24 (68.6%)	0.15	0.2
Medicaid	13 (37.1%)	6 (17.1%)		
Private insurance	5 (14.3%)	5 (14.3%)		
VL suppressed at time of last paediatric visit	17 (51.5%) [33]	9 (27.3%) [33]	0.04	0.2
Age at last paediatric visit	24.2 (1.3)	22.3 (1.4)	<0.001	1.2
Age at first adult visit	24.6 (1.16)	23.8 (1.5)	0.017	0.6
Months between last paediatric and first adult visit (linkage time)	3.8 (3.9)	18.9 (8.3)	<0.001	2.3
CD4 count at time of last paediatric visit (cells/mm^3^)	407 (309)	387 (277) [34]	0.78	0.1
Retained in first year of adult care	32 (91.4%)	30 (85.7%)	0.45	0.1
Retained in second year of adult care	22 (64.7%) [34]	16 (47.1%) [34]	0.14	0.2
VL suppressed at time of last adult visit	19 (54.3%)	12 (35.3%) [34]	0.11	0.2
CD4 count at time of last adult visit (cells/mm^3^)	406 (316)	299 (292) [34]	0.15	0.3

Values are reported as frequency (%) for categorical variables and mean (standard deviation) for continuous variables. ^a^Fisher's exact test for gender and race; Chi‐square test for all other categorical variables; two‐sample *t*‐test for continuous variables. ^b^Cohen's *w* reported for categorical variables and Cohen's *d* reported for continuous variables. VL: viral load

### Predictors of engagement outcomes

Age at last paediatric visit was significantly associated with successful linkage within three months (RR: 1.57; confidence interval (CI): 1.19–2.07; *p* = 0.001). No other demographic or clinical factors emerged as statistically significant predictors of successful linkage to care within three months of the last paediatric appointment (Table 4). However, viral load suppression and CD4+ T cell count at the last paediatric visit did appear to have a relationship with three‐month linkage that trended towards significance. Patients who had viral load suppressed at time of last paediatric visit were 76% more likely to have successfully linked in three months (46%) compared to those who did not have viral load suppression (26%; RR: 1.76; 95% CI: 0.91–3.4; *p* = 0.09). For each 50‐cell/m^3^ increase in CD4 count at time of last paediatric visit, the likelihood of successful linkage in three months increased by 3% (RR: 1.03; 95% CI: 0.99–1.08; *p* = 0.15).

**Table 4:** Predictors of linkage within three months (*N* = 72)

	Three‐month linkage		Relative risk	95% confidence interval	*p*
Sex at birth	Male	Female	1.41	0.65–3.1	0.74
	18/49 (37%)	6/23 (26%)			
Race	Black	Other	1.71	0.29–10.2	0.35
	23/67 (34%)	1/5 (20%)			
Mode of acquisition	Sexual	Perinatal	1.98	0.54–7.3	0.30
	22/61 (36%)	2/11 (18%)			
Insurance status	Insured	Uninsured	1.18	0.62–2.3	0.61
	11/30 (37%)	13/42 (31%)			
VL suppressed at time of last paediatric visit^a^	Suppressed	Not Suppressed	1.76	0.91–3.4	0.09
	12/26 (46%)	11/42 (26%)			
Age at last paediatric visit			1.57	1.19, 2.07	0.001
CD4 count at time of last paediatric visit^b^ per 50‐cell/mm^3^ increase			1.03	0.99–1.08	0.15

VL: viral load ^a^Unavailable for four patients. ^b^Unavailable for one patient.

Retention in second year of adult care was also considered as an outcome (Table 5). Increasing length of time between the last paediatric and first adult visit was associated with decreased likelihood of retention two years into adult care (RR: 0.92 per three‐month time increase; 95% CI: 0.85–0.99; *p* = 0.03), as was age at last paediatric visit (RR: 1.16; 95% CI: 1.00–1.34; *p* = 0.047). Virologic suppression at last paediatric visit showed a non‐significant trend towards predicting retention in this model: 68% of patients virologically suppressed were retained compared to 49% of those not suppressed (*p* = 0.15). Mode of acquisition and gender were also non‐significantly associated with retention, with 68% of females retained (versus 50% of males; *p* = 0.13) and 73% of perinatally infected youth retained (versus 53% of sexually infected youth; *p* = 0.15).

**Table 5:** Predictors of retention in second year of adult care (*N* = 68)

	Retention		Relative risk	95% confidence interval	*p*
Sex at birth	Male	Female	0.73	0.49–1.10	0.13
	23/46 (50%)	15/22 (68%)			
Race	Black	Other	0.93	0.44–1.96	0.84
	35/63 (56%)	3/5 (30%)			
Mode of acquisition	Sexual	Perinatal	0.72	0.47–1.12	0.15
	30/57 (53%)	8/11 (73%)			
Insurance status	Insured	Uninsured	0.98	0.64–1.50	0.92
	16/29 (55%)	22/39 (56%)			
VL suppressed at time of last paediatric visit^a^	Suppressed	Not suppressed	1.40	0.92–2.12	0.12
	17/25 (68%)	19/39 (49%)			
CD4 count at time of last paediatric visit^b^ per 50‐cell/mm^3^ increase			1.02	0.99–1.06	0.26
Three‐month linkage	Linked	Not linked	1.28	0.84–1.93	0.25
	15/23 (65%)	23/45 (51%)			
Six‐month linkage	Linked	Not linked	1.43	0.94–2.16	0.09
	19/28 (68%)	19/40 (48%)			
Age at last paediatric visit			1.16	1.00–1.34	0.047
Time between last paediatric and first adult visit per three‐month increase			0.92	0.85–0.99	0.03

^a^Unavailable for four patients. ^b^Unavailable for one patient. VL: viral load.

Finally, we examined predictors of viral load suppression after transition, using the last available viral load measurement within the two‐year window (Table 6). Viral load suppression at the last paediatric visit and linkage to adult care within three months significantly predicted virologic suppression at the most recent measurement. Patients who had viral load suppression at the time of their last paediatric visit were more than twice as likely to have viral load suppressed at their most recent adult clinic visit compared those who were not suppressed in paediatric clinic (RR: 2.31; 95% CI: 1.34–3.95; *p* = 0.002), and patients who successfully linked to adult care within three months were 76% more likely to have viral load suppressed at their most recent adult clinic visit compared to patients who did not link in three months (RR: 1.76; 95% CI: 1.07–2.9; *p* = 0.03).

**Table 6:** Predictors of most recent adult viral load suppressed (*n* = 69)

	VL suppressed		Relative risk	95% confidence interval	*p*
Sex at birth	Male	Female	0.98	0.56–1.72	0.95
	21/47 (45%)	10/22 (46%)			
Race	Black	Other	0.73	0.34–1.57	0.42
	28/64 (44%)	3/5 (60%)			
Mode of acquisition	Sexual	Perinatal	0.99	0.49–2.00	0.97
	26/58 (45%)	5/11 (46%)			
Insurance status	Insured	Uninsured	0.87	0.51–1.50	0.62
	12/29 (42%)	19/40 (48%)			
VL suppressed at time of last paediatric visit^a^	Suppressed	Not suppressed	2.31	1.34–3.95	0.002
	18/26 (69%)	12/40 (30%)			
CD4 count at time of last paediatric visit^b^ per 50‐cell/mm^3^ increase			1.02	0.99–1.04	0.20
Three‐month linkage	Linked	Not linked	1.76	1.07–2.90	0.03
	15/24 (63%)	16/45 (36%)			
Six‐month linkage	Linked	Not linked	1.47	0.88–2.47	0.14
	16/29 (55%)	15/40 (38%)			
Age at last paediatric visit			1.02	0.87–1.2	0.78
Time between last paediatric and first adult visit *per three‐month increase*			0.92	0.83–1.01	0.09

^a^Unavailable for three patients. ^b^Unavailable for one patient. VL: viral load.

## Discussion

In this retrospective analysis of transition among HIV‐infected youth in our centre, rates of eventual linkage to care were high, but with a very wide range of times between the last paediatric and the first adult visit. Rates of retention in adult care were similarly high when assessed one year after transition (either formal transition after being enrolled in paediatric care or de facto transition after experiencing a gap in care); however, they dropped off significantly by the two‐year mark. Rates of virologic suppression were low in this cohort and did not differ significantly between the paediatric and adult settings; however, those who were not virologically suppressed in paediatrics were less likely to be become engaged after transitioning to the adult clinic.

It is notable that age at last paediatric visit, age at first adult visit and time between the two visits emerged as significant predictors of adult clinic engagement in several analyses; however, these statistical associations likely reflect inherent confounding of these variables with the outcomes in question, as opposed to a causative effect of age (or associated developmental maturity) itself. At the IDP, transition occurs almost exclusively based on age (although the cutoff changed over time) ‐ so the finding that those who experienced a gap in care were younger at last paediatric visit, for example, simply reflected that these patients did not stay enrolled until the time when a formal transition would have occurred; the same can be said for the finding that age at last paediatric visit was associated with poorer linkage within three months.

Taken together, our findings suggest that there are high rates of disengagement among YLHIV even while they are still in paediatric care, often before healthcare transition can even be planned or initiated. Such disengagement, as well as incomplete retention or virologic control while in the paediatric setting, portends poor odds of successful engagement in adult care after transition. Optimizing transition may therefore require earlier planning in the paediatric clinic, as well as supporting youth beyond the initial linkage to (and potentially, beyond the first year in) the adult clinic.

Our findings about the difficulties achieving timely linkage are particularly disheartening, given the consideration that the paediatric and adult clinics being studied are located in the same physical building. Potential explanations for these challenges include differences in support between the two clinics as well as attachment to paediatric staff/fear of adult clinic that have been outlined in multiple prior qualitative studies [6,20]. Additionally, many of these patients became disengaged from care before they reached an age at which transition discussions would have been initiated. The gap in care for these patients may be attributable to life stressors external to the clinic setting.

Our analysis was somewhat unusual in that we included data from patients who were enrolled in paediatric care immediately prior to transition, as well as those who experienced a gap in paediatric care and returned to re‐enrol in the clinic as adults (and thus were transitioned de facto). Although one could argue that these are two very different groups, we would argue that this brings up a critical point that is missing from much of the healthcare transition literature. Existing protocols and guidelines in this field focus on patient readiness and the physician‐to‐physician handoff between paediatricians and internists around the time of the transition [2,5,21]. Unfortunately, this approach ignores that patients must be previously engaged in care to be intentionally transitioned in the first place. Those who are not engaged will still undergo transition by necessity when they become too old to be seen by paediatricians, but will undergo this process with even less guidance and support than their better engaged peers. This points to the need to expand social services and intervention programmes greatly and to include high‐risk YLHIV in transition planning even before such a move becomes imminent (e.g. with structured protocols for transition support that begin years before a transition is planned). Such an approach could be counterintuitive for clinicians, who may be in the position of deferring discussion of transition for patients who are medically or socially unstable. However, our findings about the transition difficulties among these youth suggest the need for engagement interventions that have a much wider scope than the sometimes seemingly narrow transition window.

Given the poor prognostic implications of detectable viremia in paediatric clinic, our findings also suggest a role for directing intensive transition support towards these youth. As all such clinics are contending with problems of resource limitation, intensive case management, peer navigation and other interventions may not be feasible for all youth undergoing transition. Our findings suggest that directing these resources towards those who are unable to achieve viral suppression in paediatrics might be a strategy for utilizing these resources in the highest yield manner possible.

### Limitations

Our study has several limitations. Our sample size, although larger than other studies in this field, was still too small to answer some of our questions, as indicated by several findings that approached, but did not reach statistical significance. We utilized a retrospective design and had incomplete information about many patients. By excluding those who never had an appointment scheduled in the adult clinic, we biased our sample in favour of those who may have been relatively more engaged. On the other hand, we cannot exclude that those patients who disengaged from care in our clinic may have received care elsewhere, and this gap could have overestimated disengagement. The IOM definition of retention, though widely used, is a very non‐specific measure of engagement. Use of another retention metric such as missed appointments may have given us a more nuanced picture of our patients’ engagement patterns; unfortunately, we were unable to document missed appointments from those patients whose records were in paper charts (where missed appointments were not recorded) as opposed to the EMR. Finally, due to limitations in our data set, we were not able to include about specific antiretroviral regimens or even generally whether or not a patient was prescribed HIV medication. Such information would have served to help contextualize the rates of viral suppression in our cohort, which may be lower than current rates due to improved antiretroviral therapy.

## Conclusions

In spite of these limitations, our study represents one of the largest cohorts of YLHIV transitioning from paediatric to adult care to date. We plan to incorporate these findings into the development of structured, comprehensive protocol to support healthcare transition within the IDP. Future studies would benefit from a prospective, longitudinal design to help attenuate some of the biases described above. Mixed‐methods approaches that involve YLHIV would be particularly useful to gain specific, practical suggestions to inform future strategies for supporting engagement before, during and after healthcare transition. Most importantly, our findings support the development, testing and implementation of comprehensive interventions to improve engagement among youth who are still in paediatric care, as these are likely to have the potential for further downstream effects on engagement through the transition and to care in adult‐oriented settings.

## Competing interests

The authors have no competing interests to declare.

## Authors’ contributions

S.A.H. conceived the study and drafted the manuscript. R.C. contributed to study conception, interpretation of data and manuscript revisions. A.K. contributed to data analysis and revising the manuscript. A.C.G. contributed to interpretation of data and manuscript revisions. E.H. contributed to data analysis and revising the manuscript. R.S. contributed to study conception and revising the manuscript. C.D.R. contributed to study conception, interpretation of data and revising the manuscript. All authors have read and approved the final version of the manuscript prior to submission.

## Acknowledgements

We would like to acknowledge the work of graduate research assistants Sonam Patel, Zelalem Adefris and Emily Grossniklaus, who assisted with data abstraction.

## Funding

This work was supported by a grant from the Emory Medical Care Foundation to S.A.H. S.A.H.'s work on this manuscript is also supported by the Harold Amos Medical Faculty Development Program of the Robert Wood Johnson Foundation [Grant# 73309].
